# HIF-1α promotes ZEB1 expression and EMT in a human bladder cancer lung metastasis animal model

**DOI:** 10.3892/ol.2021.12860

**Published:** 2021-06-10

**Authors:** Jianning Zhu, Zhixin Huang, Mengzhao Zhang, Weiyi Wang, Hua Liang, Jin Zeng, Kaijie Wu, Xinyang Wang, Jer-Tsong Hsieh, Peng Guo, Jinhai Fan

Oncol Lett 15: 3482-3489, 2018; DOI: 10.3892/ol.2018.7764

Subsequently to the publication of the above article, an interested reader drew to the authors’ attention that Fig. 2C and 2D apparently contained overlapping panels, suggesting that these data for purportedly different experiments had been derived from the same original source. The authors re-examined their original data, and realized that the errors arose inadvertently during the process of compiling the figure. Essentially, multiple photographs of random fields in two directions per sample had been captured, and one photograph of the T24-P group in a different direction was accidentally mis-used for the T24-L/siHIF-1α group in Fig. 2C; another photograph of the T24-P group in a different direction was accidentally mis-used for the T24-L group in Fig. 2D.

A corrected version of Fig. 2 is shown below, featuring the corrected data panels for Fig. 2C and D. Note that the errors in the Figure did not affect either the results or the conclusions reported in this study. The authors are grateful to the Editor of *Oncology Letters* for granting them the opportunity to publish this corrigendum, and regret any inconvenience caused to the readership of the Journal.

## Figures and Tables

**Figure 2.**
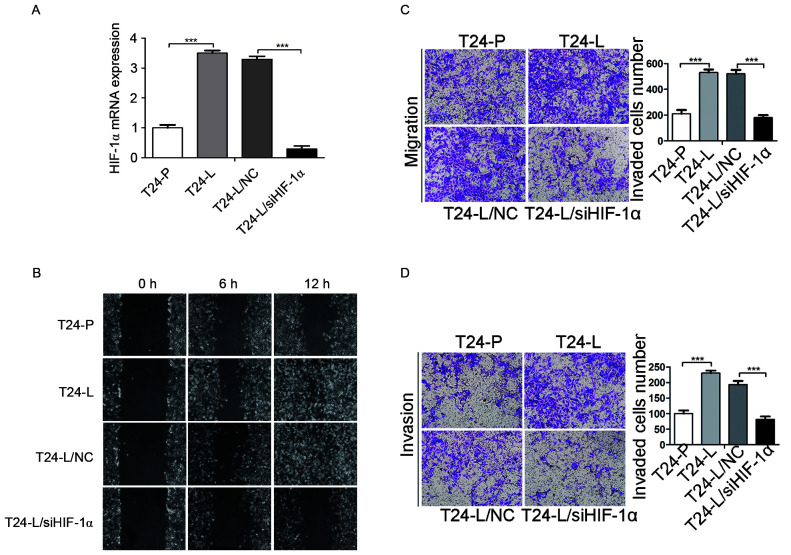
Knockdown of HIF-1α by siRNA reduces the malignancy of T24-L cells. (A) Reverse transcriptase-quantitative polymerase chain reaction was used to demonstrate the expression of HIF-1α in T24-L and T24-P, and the efficiency of siRNA against HIF-1α in T24-L. The expression of HIF-1α is significantly increased in T24-L. ***P<0.001 si HIF-1α vs. NC. (B) Wound-healing assay to demonstrate that T24-L cells exhibited higher migration activity than T24-P cells, and T24-L control cells exhibited higher migration activity compared with HIF-1α knockdown T24-L cells. Boyden chamber assays to demonstrate that (C) the migration and (D) the migration and invasion ability of T24-L cells was greater compared with T24-P cells, and that HIF-1α knockdown impaired the migration and invasion capacity of T24-L cells. Magnification, ×100. ***P<0.001. HIF-1α, hypoxia inducible factor-1α; siRNA, small interfering RNA; T24-L,T24 lung metastasis cells; T24-P, T24 parental cells; NC, negative control.

